# Wnt Signaling in Vascular Calcification

**DOI:** 10.3389/fcvm.2021.708470

**Published:** 2021-09-14

**Authors:** Kaylee Bundy, Jada Boone, C. LaShan Simpson

**Affiliations:** Agricultural and Biological Engineering, Mississippi State University, Starkville, MS, United States

**Keywords:** vascular calcification, Wnt signaling, phenotypes, transdifferentiation, Runx2

## Abstract

Cardiovascular disease is a worldwide epidemic and considered the leading cause of death globally. Due to its high mortality rates, it is imperative to study the underlying causes and mechanisms of the disease. Vascular calcification, or the buildup of hydroxyapatite within the arterial wall, is one of the greatest contributors to cardiovascular disease. Medial vascular calcification is a predictor of cardiovascular events such as, but not limited to, hypertension, stiffness, and even heart failure. Vascular smooth muscle cells (VSMCs), which line the arterial wall and function to maintain blood pressure, are hypothesized to undergo a phenotypic switch into bone-forming cells during calcification, mimicking the manner by which mesenchymal stem cells differentiate into osteoblast cells throughout osteogenesis. RunX2, a transcription factor necessary for osteoblast differentiation and a target gene of the Wnt signaling pathway, has also shown to be upregulated when calcification is present, implicating that the Wnt cascade may be a key player in the transdifferentiation of VSMCs. It is important to note that the phenotypic switch of VSMCs from a healthy, contractile state to a proliferative, synthetic state is necessary in response to the vascular injury surrounding calcification. The lingering question, however, is if VSMCs acquire this synthetic phenotype through the Wnt pathway, how and why does this signaling occur? This review seeks to highlight the potential role of the canonical Wnt signaling pathway within vascular calcification based on several studies and further discuss the Wnt ligands that specifically aid in VSMC transdifferentiation.

## Introduction

Cardiovascular disease (CVD) is a worldwide epidemic. As the leading cause of death across the world, many researchers are working to better understand the causes and mechanisms resulting in the disease (1). One of the contributors and predictors of cardiovascular mortalities is vascular calcification, the buildup of hydroxyapatite deposits within the arterial wall. The mineral deposition can occur in either the intimal or medial layers of the arteries; the location depends upon the causative factors and can result in different effects within the body (2). Conditions conducive of calcification include, but are not limited to, hypercalcemia, hyperphosphatemia, and mechanical stress which induce changes to the arteries on a cellular level. Medial calcification, which occurs within the vascular smooth muscle cells (VSMCs) lining the middle layer of the arterial wall, has been linked to hypertension, stiffness, and increased risk of heart failure (3). A comprehensive understanding of the various changes within the VSMCs during calcification may help to identify a key regulator to target with a treatment. VSMCs are derived from mesenchymal stem cells and are not terminally differentiated; they are known to maintain their plasticity and can differentiate into other mesenchymal cell derivatives (4). During calcification, studies have shown VSMCs undergo a cellular mediated phenotypic switch into cells resembling bone forming osteoblasts, characterized by a loss of smooth muscle markers and an upregulation of osteogenic markers (5). Runx2, a transcription factor necessary for osteoblast differentiation, is upregulated within calcifying VSMCs and may be the cause of this transdifferentiation (6). Runx2 is a target gene of the Wnt signaling cascade, which is known to regulate bone development during embryogenesis, as well as direct bone turnover and remodeling (7). Because mineral deposition in VSMCs appears to be very similar to bone formation, many studies have begun investigating Wnt signaling as a possible mechanism and regulator of vascular calcification. This mini review seeks to culminate the observations made so far on VSMC phenotypes and the regulatory role of Wnt in VSMC plasticity to help construct a holistic but focused view of the cellular factors influencing vascular calcification.

## VSMC Phenotypes

**Figure 1:**
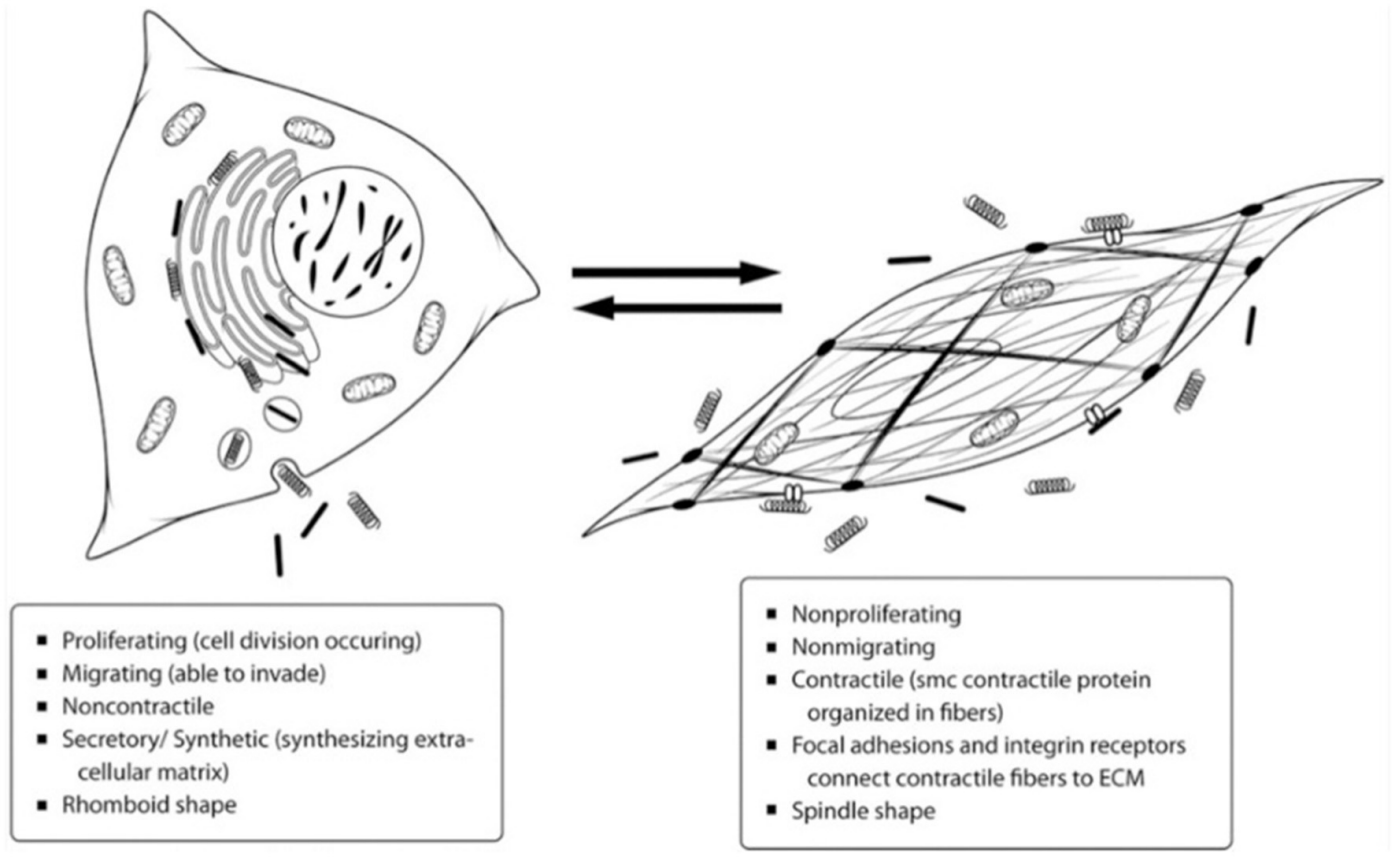
Depiction of the differences between the contractile and synthetic VSMC phenotypes. Most VSMCs exist in the contractile state on the right to maintain blood pressure, but they can dedifferentiate into the synthetic phenotype on the left as necessary for proliferation and tissue repair (12).

Before the 1960s, it was commonly thought that there were two cell types in the arterial media: smooth muscle cells and fibroblasts. The reason for this assumption was due to the presence of connective tissue within the middle layer, like that formed by fibroblasts. However, in the early 1960s, studies began providing evidence for only one cell type, smooth muscle cells, within the arterial media (8, 9). In 1967, Wissler suggested that the medial cells are a multifunctional mesenchyme cell type capable of both contracting and fabricating connective tissue. Wissler was one of the first to realize smooth muscle cell plasticity and that the once thought fibroblasts are really a dedifferentiated smooth muscle cell (10). Over the next few decades, much more was learned about the range of VSMC phenotypes. The principal function of VSMCs within the body is to maintain blood pressure. To achieve this function, the cells primarily maintain a contractile phenotype that is characterized by slow proliferation, response to neurotransmitters, and expression of cellular markers such as α-smooth muscle actin, smooth muscle myosin heavy chains 1 and 2, calponin, and smoothelin (4). When in the contractile state, the cell has a spindle shape with many focal adhesions and integrin receptors to connect the cell to the ECM and allow for contraction. The cytoplasm contains primarily myofilaments, with a low number of other organelles such as rough endoplasmic reticulum, Golgi, and free ribosomes (11). In response to a necessary change in function, such as the need for increased proliferation, VSMCs may display a dedifferentiated phenotype commonly referred to as synthetic. The synthetic state is characterized primarily by proliferation, migration, and extracellular matrix production, so the cytoplasm of the cells contains a greater amount of rough endoplasmic reticulum, Golgi, and free ribosomes and a smaller number of myofilaments (11). The distinctions between the contractile and synthetic phenotypes can be seen in Figure 1 (12). The need for greater proliferation as seen in the synthetic state can arise in various circumstances but has been particularly characterized in response to vascular injury.

## VSMC Transdifferentiation

Following injury and for vascular repair to occur, the VSMCs revert to the synthetic state explained above. However, as the synthetic phenotype occurs as a dedifferentiated state, the cells can then further differentiate into other cell types depending on environmental cues. VSMCs have been shown to downregulate contractile proteins and display characteristics of other mesenchymal lineage cell types, including those of osteoblasts, chondrocytes, and adipocytes (13). In pro-calcifying conditions, such as high levels of serum phosphate, VSMCs begin to express osteogenic markers including Runx2, Sp7, osteopontin, osteocalcin, alkaline phosphatase, Sox9, and collagen types II and X (4). A study done by Patel et al. sought to evaluate the similarity between bone formation and calcification by comparing mouse osteoblast with control and calcifying VSMCs (14). The quantity of calcium deposition between osteoblasts and calcifying VSMCs was similar, but osteoblasts formed many large bone nodules whereas calcifying VSMCs formed small discrete regions of calcification. Calcifying VSMCs saw a 6-fold increase in early osteoblast markers Runx2 and Sp7 compared to control VSMCs but still a 3-fold lower amount compared to the osteoblasts. The study concluded that calcifying VSMCs take on a transitional phenotype between but distinct from that of healthy VSMCs and bone-forming osteoblasts (14). Many other studies have also noted the increase and possible requirement of Runx2 expression, a transcription factor necessary for osteoblast differentiation, in calcifying VSMCs (15–18). Though it is well understood that Runx2 is at least partially responsible for the osteogenic switch, it is necessary to determine why the transcription factor is being upregulated in the cells. Gaur et al. determined the Runx2 gene is directly targeted by the canonical Wnt signaling pathway which activates the gene and regulates bone production during development and in adults (19). Because of the governing role of Runx2 in osteoblast differentiation and vascular calcification, studies are investigating Wnt signaling as a possible mechanism of calcification.

## The WNT Signaling Cascade

**Figure 2:**
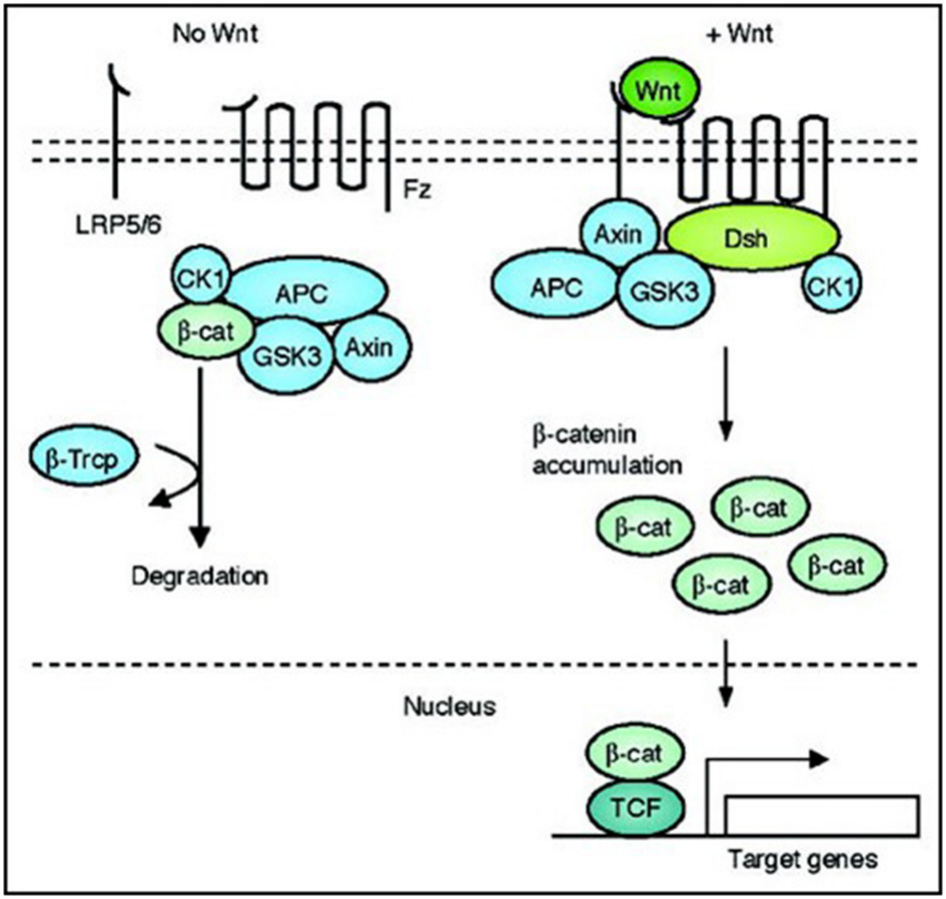
Schematic of the canonical Wnt signaling cascade. Binding of a Wnt ligand to the cell surface causes the translocation of β-catenin to the nucleus and the upregulation of Wnt target genes (20).

A family of 19 secreted glycoproteins, Wnt signaling is conserved among metazoan animals to regulate many cellular functions during development including cell fate determination, migration, polarity, primary axis formation, organogenesis, and stem cell renewal (20). Wnt ligands each consist of 350-400 amino acids including 22–24 conserved cysteine residues (7). The signaling cascade is activated when one of the extracellular Wnt ligands binds to a Frizzled (Fz) receptor. Fz is a family of 10 different seven-member transmembrane proteins. A Wnt signal binds to the cysteine-rich extracellular N-terminal of a Fz receptor associated with co-receptors such as LRP5 and LRP6 of the low-density lipoprotein receptor family (20). LRP5 and LRP6 are transmembrane proteins that help Fz to induce the canonical Wnt pathway (7). After Wnt binds to Fz, the signal recruits the cytoplasmic phosphoprotein Disheveled (Dsh) to the plasma membrane. At this point, the signaling pathway diverges into three separate branches: Canonical (β-catenin dependent), Planar Cell Polarity, and Wnt/Ca^2+^ (20). The canonical branch has been most widely studied and is the segment hypothesized to play a role in vascular calcification. Dsh aids in the recruitment of an Axin and GSK3 complex (21). Under normal, non-activated Wnt conditions, Axin is the scaffolding protein of a β-catenin destruction complex. The destruction complex is made up of Axin, glycogen synthase kinase 3 (GSK-3), casein kinase 1 (CK1), adenomatous polyposis coli (APC) protein, and the E3-ubiquitin ligase β-TrCP (22). The complex typically phosphorylates and proteolytically degrades any accumulation of β-catenin in the cytoplasm. When Axin is recruited to the plasma membrane because of Wnt signaling, the destruction complex is disassembled, resulting in an upregulation of β-catenin (21). β-catenin then translocates to the nucleus and forms a transcriptional complex with LEF-1/TCF DNA-binding transcription factors. The complex associates to the promoter of Wnt target genes that results in the upregulation of those genes (20). In summary, during canonical Wnt signaling, the binding of Wnt ligands to the cell membrane inhibits the β-catenin destruction complex resulting in the translocation of β-catenin to the nucleus where the transcription of Wnt target genes is induced, as shown in Figure 2 (20).

## WNT and RUNX2 in Osteogenesis

As stated before, studies have shown that Runx2 is a target gene of Wnt signaling, and activation of Runx2 by Wnt stimulates osteoblast differentiation and bone formation (19). In mesenchymal stem cells, Runx2 regulates the expression of other bone related proteins, such as osterix, osteocalcin, and sclerostin, directing the cell to an osteogenic phenotype (6). Expression of Runx2 begins in uncommitted stem cells, increases in osteoblast precursors, peaks in immature osteoblast, and decreases once osteoblasts mature (23). This expression of Runx2 is modulated by canonical Wnt signaling, resulting in an inhibition of chondrocyte differentiation in early mesenchymal cells and directing the progenitors to become osteoblasts (23). The process occurs during embryonic development when establishing the body axis and tissue and organ development and functions after birth in bone maintenance and repair (24).

## WNT in Vascular Calcification

Because Wnt is involved in bone turnover and calcifying smooth muscle cells resemble osteoblasts, Wnt may play a governing role in calcification. A recent comprehensive review by Tyson et al. highlights the role of mechanotransduction, explaining how the compressive and tensile strains experienced by VSMC under increased stress may induce bone-like Wnt mediated remodeling in the arterial wall (25). Interestingly, Wnt ligands and signals are found in noncalcifying VSMCs and may contribute to normal regulation of the VSMC phenotype, proliferation, and survival, particularly in response to vascular injury. Studies from the early 2000s indicate the presence of Wnt proteins in VSMCs. A study done in 2004 by Wang et al. used a transfection assay to show that loss of the Wnt coreceptor LRP6 function in VSMCs inhibited cell cycle progression, demonstrating a role for LRP6 and Wnt in VSMC growth and fate (26). Another study done by Wang et al. in 2005 noted that Fz1 is highly expressed in VSMC tissues indicating active Wnt signaling, and they suggest a role in development and response to environmental stimulus (27). In agreement with these studies, in 2014 Wu et al. found that a Wnt ligand influences VSMC migration and adhesion to collagen type I (28). A transwell migration and wound healing assay showed that Wnt3a significantly increased VSMC migration, and an adhesion assay showed that Wnt3a treated VSMCs were able to adhere more to collagen type I. The study also used Western blot analysis to test for several canonical Wnt components, including β-catenin, GSK-3β, ILK, and β1-integrin. Wnt3a treatment upregulated phosphorylated β-catenin, phosphorylated GSK-3β, and ILK and activated β1-integrin in VSMCs, providing additional evidence of Wnt effecting protein expression and potential therapeutic targets (28).

Other studies have looked at the involvement of Wnt particularly in calcifying VSMCs. Mikhaylova et al. found that Wnt3a induced a 3.5-fold increase in mineralization when used with hypertrophic chondrocyte conditioned media, suggesting Wnt paired with other chondrocyte-derived factors, such as VEGF, may be a positive regulator of calcification (29). Rong et al. used phosphate and BMP-2 to induce calcification in VSMCs, and then tested the cultures for various cellular markers (5). They were able to observe a phenotypic change as SM 22α and α-SMA were downregulated and osteogenic markers including Msx2, RunX2, Pit1, and β-catenin were upregulated. When β-catenin was knocked down using a transfection assay, these results were reversed, indicating a dependent role of β-catenin and Wnt signaling in VSMC transdifferentiation (5). In 2016, Cai et al. also provided strong evidence for the involvement of Wnt signaling in VSMC transdifferentiation and mineralization (6). In a pro-calcifying high phosphate environment, RunX2 expression was induced in a time-dependent manner, observed by Western blot analysis. To determine if the increase in RunX2 expression was caused by Wnt signaling, they used Western blots to track β-catenin activity and found that both dephosphorylated and phosphorylated β-catenin was upregulated. Immunostaining showed the high phosphate treatment also promoted β-catenin translocation to the nucleus, a typical cellular response to Wnt signaling, and they were able to use luciferase reporter assays to identify two specific TCF binding elements that mediate the interaction with TCF in response to β-catenin translocation. Further evidence of Wnt included a positive result for phosphorylation of LRP6, a Wnt dependent reaction. To confirm these results, a Wnt inhibitor was used that abolished RunX2 induction during high phosphate treatment. VSMCs were then treated with Wnt3a, which successfully induced calcium deposition and osteocalcin induction. The results from Cai et al. suggest a specific pathway in which Wnt3a can activate β-catenin and induce RunX2 and osteocalcin expression and promote calcification of VSMCs (6).

## Which WNT?

Though there is surmounting evidence of the involvement of Wnt in vascular calcification, each study identifies different inducers, inhibitors, and regulatory proteins relative to Wnt and even other signaling pathways, such as VEGF and BMPs mentioned previously. Although they must be studied independently, it is likely that each of these different processes may contribute and work together to create an environment conducive to VSMC transdifferentiation and thus calcification. As Wnt has been more heavily investigated as a possible mechanism, some studies have begun testing specific Wnt ligands to determine if one contributes most significantly toward calcification. Wnt7b has been shown to play a role in vascular development and can activate canonical Wnt signaling (27). Wnt16 is also expressed in VSMCs. In a recent study by Behrmann et al., *in vitro* and *in vivo* studies showed that Wnt16 suppressed the contractile phenotype, supported osteofibrogenic matrix metabolism, and contributed to aortic stiffening (30). However, the most studied Wnt ligand in vascular calcification is Wnt3a. Some of these studies were referenced in the previous section (6, 28, 29). These studies provide some of the most convincing support for the Wnt signaling cascade in calcification indicating that Wnt3a may be the focus of future studies working toward a treatment.

## Conclusion

In conclusion, VSMCs display a range of phenotypes within two key states: contractile and synthetic. When VSMCs exist in the synthetic state, they exhibit a less differentiated phenotype and can be directed down different cell lineages in response to abnormal environmental cues. A high phosphate environment can induce calcification in VSMCs and direct them toward an osteogenic phenotype. This transdifferentiation is characterized by a loss of VSMC markers and an increase in osteogenic markers, most notably RunX2. Because of the upregulation of RunX2, many believe the canonical Wnt signaling pathway may be the cellular mechanism resulting in the osteogenic switch of VSMCs. RunX2 is a target gene of Wnt, which also involves many other proteins, including β-catenin, LRPs, Fzd, Dsh, and Axin. Wnt has been found to regulate normal VSMC phenotype and proliferation, and much data has been elucidated for a role in VSMC transdifferentiation during calcification. Many studies have used Western blots, PCR, and luciferase assays to identify various Wnt proteins, notably β-catenin, and track the Wnt cascade to determine its involvement in the initiation and regulation of medial vascular calcification. Future research strategies should focus these methods on regular time intervals to determine at what point during VSMC transdifferentiation Wnt is activated. Experiments should also begin investigating other Wnt proteins in addition to β-catenin to identify any other major regulatory factors. If a particular fate-determining time point and feedback loop is identified as the primary mechanism of action for VSMCS transdifferentiation, Wnt could be manipulated as a potential therapy for vascular calcification. Knocking out Wnt, or its regulators, with Wnt inhibitors, such as sclerostin, and determining the effect will help determine whether Wnt is a valuable therapeutic target. Additional studies should be done like McArthur et al. who found that sclerostin could successfully prevent Wnt proteins from attaching to their corresponding receptors and ultimately resulted in reduction of calcification (16). The study illustrates the potential of manipulating Wnt for calcification treatment, though much more extensive preclinical trails are necessary. The most common preclinical disease model for atherosclerosis, which closely mimics calcification, is Apoe^−/−^ mice (31). More recently, a model utilizing calcium chloride to induce calcification has been established (32). Using these models to further test Wnt targeted calcification treatments may be a logical next step. This literature review has summarized the current understanding of the role of VSMCs in calcification and helped to identify ways in which to further study the mechanism of the disease. However, there are some limitations and current gaps in knowledge. Though Wnt must be isolated to study the pathway independently, more than likely the Wnt cascade operates dependently upon other pathways and external factors. In addition, to our knowledge there has not yet been significant investigation into the presence of Wnt ligands under normal conditions, and it is unclear whether Wnt is always present within the arterial media. Furthermore, if this is the case, studies have not explored what activates Wnt signaling leading to calcification. As the Wnt family consists of extracellular ligands, cell-to-cell signaling and mechanical stimulus could be contributors. Thought it appears targeting Wnt could be a potential treatment, it is yet unclear whether a Wnt therapy would reverse calcification or only prevent further buildup. Based on these theories, there remains a need for investigating further into the typical behavior of Wnt under healthy and disease conditions, as well as the underlying mechanism behind the activation of the Wnt cascade. As a result, additional research may lead to preclinical trials and eventually allow for a targeted treatment for vascular calcification.

## Author Contributions

KB conducted a comprehensive literature review and transcribed the bulk of the manuscript. JB contributed to the literature review and writing of the manuscript. CS helped to develop the concept for the work and played a supervisory and editorial role. All authors contributed to the article and approved the submitted version.

## Funding

This work was supported by the Mississippi Agricultural and Forestry Experiment Station.

## Conflict of Interest

The authors declare that the research was conducted in the absence of any commercial or financial relationships that could be construed as a potential conflict of interest.

## Publisher's Note

All claims expressed in this article are solely those of the authors and do not necessarily represent those of their affiliated organizations, or those of the publisher, the editors and the reviewers. Any product that may be evaluated in this article, or claim that may be made by its manufacturer, is not guaranteed or endorsed by the publisher.
